# scL-2PAM: A Novel Countermeasure That Ameliorates Neuroinflammation and Neuronal Losses in Mice Exposed to an Anticholinesterase Organophosphate

**DOI:** 10.3390/ijms25147539

**Published:** 2024-07-09

**Authors:** Manish Moghe, Sang-Soo Kim, Miaoyin Guan, Antonina Rait, Kathleen F. Pirollo, Joe B. Harford, Esther H. Chang

**Affiliations:** 1 Department of Oncology, Lombardi Comprehensive Cancer Center, Georgetown University Medical Center, Washington, DC 20057, USA; mmm393@georgetown.edu (M.M.); raita@georgetown.edu (A.R.); pirollok@georgetown.edu (K.F.P.); 2 Department of Biochemistry and Molecular & Cellular Biology, Georgetown University Medical Center, Washington, DC 20057, USA; 3 SynerGene Therapeutics, Inc., Potomac, MD 20854, USA; harfordj@synergeneus.com

**Keywords:** organophosphate, neuroinflammation, pralidoxime, nanotherapeutic, neuronal loss

## Abstract

Due to their inhibition of acetylcholinesterase, organophosphates are among the most toxic of chemicals. Pralidoxime (a.k.a 2-PAM) is the only acetylcholinesterase reactivator approved in the U.S., but 2-PAM only poorly traverses the blood–brain barrier. Previously, we have demonstrated that scL-2PAM, a nanoformulation designed to enter the brain via receptor-mediated transcytosis, is superior to unencapsulated 2-PAM for reactivating brain acetylcholinesterase, ameliorating cholinergic crisis, and improving survival rates for paraoxon-exposed mice. Here, we employ histology and transcriptome analyses to assess the ability of scL-2PAM to prevent neurological sequelae including microglial activation, expression of inflammatory cytokines, and ultimately loss of neurons in mice surviving paraoxon exposures. Levels of the mRNA encoding chemokine ligand 2 (CCL2) were significantly upregulated after paraoxon exposures, with *CCL2* mRNA levels in the brain correlating well with the intensity and duration of cholinergic symptoms. Our nanoformulation of 2-PAM was found to be superior to unencapsulated 2-PAM in reducing the levels of the *CCL2* transcript. Moreover, brain histology revealed that scL-2PAM was more effective than unencapsulated 2-PAM in preventing microglial activation and the subsequent loss of neurons. Thus, scL-2PAM appears to be a new and improved countermeasure for reducing neuroinflammation and mitigating brain damage in survivors of organophosphate exposures.

## 1. Introduction

Organophosphates (OP) are extremely toxic chemicals that inactivate acetylcholinesterase (AChE), an enzyme responsible for removing the excitatory neurotransmitter acetylcholine (ACh) from synapses of the central nervous system and neuromuscular junctions [1,2]. The inactivation of AChE leads to hyperstimulation of cholinergic pathways and triggers excitotoxicity that can result in progressive symptoms of tremors, convulsions, seizures, respiratory distress, and death [1,3,4,5,6]. Although OP nerve agents are banned under the Chemical Weapons Convention [7], various OPs have been used historically in chemical warfare and against civilian populations [7,8]. In addition, broadly used OP pesticides like diisopropyl fluorophosphate (DFP) and parathion are associated with recurring incidences of accidental occupational or environmental exposures as well as intentional self-poisoning largely in lower-income, rural settings [9].

Long-term brain damage and neurological dysfunction has been observed in the survivors of OP exposures. Studies involving the victims of the Tokyo subway terrorist attacks and soldiers suspected to have been exposed to chemical agents in Iraq have revealed increased electroencephalogram (EEG) alterations, impaired motor function, and significant memory loss [10,11,12,13]. A 67-month post-exposure study involving the 1995 Tokyo subway survivors [14] highlighted enduring cognitive symptoms with reduced gray matter content in the right insular, right temporal, and left hippocampus and decreased white matter content in the left temporal stem. The study found a positive correlation between left subinsular white matter volume and plasma cholinesterase activities at the time of the incident. Longer-term neurological damage in US Army veterans with potential exposures to chemical agents have been reported [15]. In animal models, neuronal losses were noted after OP exposures, with consequent neurocognitive and behavioral deficits observed [4,16,17,18,19,20,21]. It appears clear that brain damage can ensue from OP exposures that result in AChE inhibition. It follows that survivors would benefit if the OP countermeasures they received were capable of mitigating these neuronal sequelae.

The inhibition of AChE by OPs leads to the accumulation of its substrate (ACh) in synapses that causes the cholinergic crisis that includes seizures that can progress to status epilepticus (SE). Seizures have been seen as a key driver in brain damage, with neuronal degeneration observed in mice that developed SE and no such degeneration seen in the mice that did not experience SE [22,23]. The path from seizures to brain damage passes through neuroinflammation [24], a multifaceted phenomenon with its major hallmark being microglial activation [25]. The activation of brain microglial cells is accompanied by an increase in expression of ionized calcium binding adaptor molecule 1 (IBA1), and increases in IBA1 expression have been observed after OP exposure [17,26,27,28]. Microglial activation is also coupled to increases in expression of a number of proinflammatory cytokines including tumor necrosis factor α (TNFα), interleukin 6 (IL6), interleukin 1β (IL1β), and CCL2. This surge in cytokine expression appears to play a critical role in neuropathology that emanates from OP exposures [20,26,29,30]. Indeed, neuroinflammation is seen as a key therapeutic target for reducing the long term neuronal consequences of OP toxicity [17,20,24,31].

The current standard medical treatment of OP poisoning involves a cholinesterase reactivator (2-pyridine aldoxime methyl chloride, a.k.a. pralidoxime or 2-PAM) combined with a muscarinic receptor antagonist (atropine sulfate), and a benzodiazepine anticonvulsant [32,33]. Depending on OP exposure levels, this treatment regimen, if administered timely, can be effective in preventing OP-caused death. However, 2-PAM does not readily cross the blood–brain barrier (BBB), and this limits its ability to reactivate OP-inactivated AChE in the CNS and to mitigate the seizures triggered by OP exposures [34,35]. We are developing a novel brain-penetrating nanoformulation of 2-PAM (termed scL-2PAM), which consists of the oxime encapsulated within a cationic liposome that is decorated with a single-chain antibody fragment (scFv) recognizing the transferrin receptor (TfR) [36]. The scL-2PAM nanocomplex is designed to cross the BBB through TfR-mediated transcytosis and thereby move its 2-PAM payload into the CNS to reach inactivated AChE in the brain. A preliminary mass spectrometry assessment of 2-PAM in the brains of mice receiving either free 2-PAM or scL-2PAM indicates higher efficiency for brain penetration of scL-2PAM than free 2-PAM, supporting our hypothesis of improved BBB crossing. Further detailed studies involving direct measurements of 2-PAM in the brain are ongoing. Our previous study of survival following paraoxon exposure, a commonly used OP challenge agent [37,38], demonstrated the superiority of scL-2PAM over free 2-PAM as a countermeasure against OP toxicity in mice [36]. We observed that scL-2PAM was more effective than free 2-PAM in ameliorating the signs and symptoms of cholinergic crisis, in reactivating paraoxon-inactivated AChE in the brain, and in improving survival rates for paraoxon-exposed mice. In the previous study [36], it was demonstrated that scL-2PAM is more effective than free 2-PAM in reactivating and preserving AChE activity in the brains of paraoxon-exposed mice. This study builds on that foundation, focusing specifically on the neuroprotective effects of scL-2PAM mediated by its reactivation of AChE. In the present study, we evaluated the effectiveness of scL-2PAM in reducing the neurological consequences observed in mice surviving paraoxon exposures. In head-to-head comparisons, we assessed the impact of the two different oxime formulations on microglial activation and neuroinflammatory signaling. Most importantly, we quantified loss of neurons after paraoxon exposures and the ability of the two different 2-PAM formulations to prevent neuronal loss. By all criteria examined, we found that scL-2PAM was superior to free 2-PAM.

## 2. Results

### 2.1. Paraoxon Exposure Induces Inflammation in the Brain

**Figure 1:**
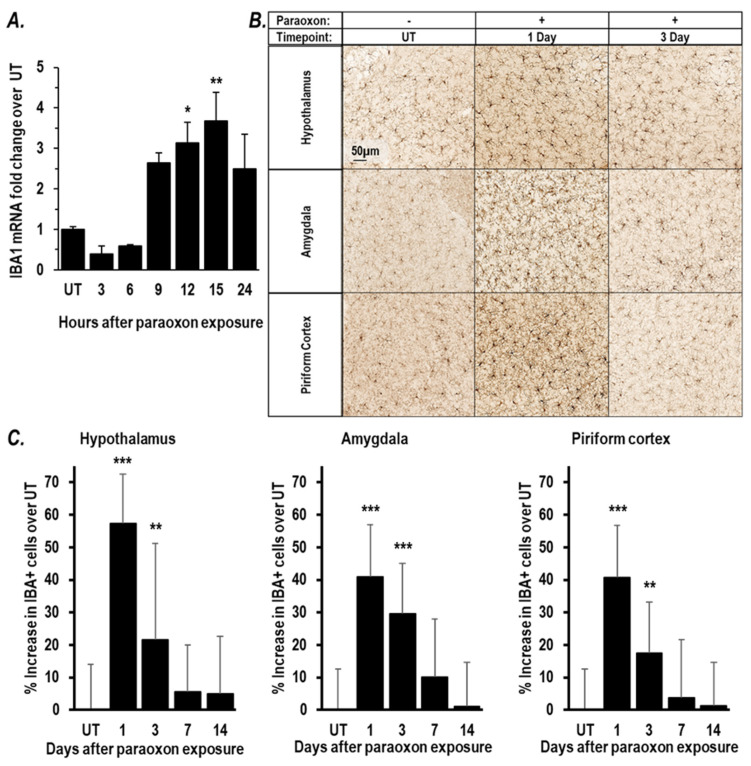
Paraoxon exposure causes microglial activation in brains of survivors. Microglial activation was assessed in the brains of paraoxon-treated mice. (**A**) RT-PCR analysis of mRNA encoding the microglial marker IBA1 in RNA isolated at indicated times after exposure to paraoxon at 0.85 × LD50 (*n* = 3/time point). IBA1 mRNA level is shown as a fold-change from the baseline values in brains from naïve mice (i.e., without paraoxon treatment). (**B**,**C**) Mice were exposed to paraoxon at 1 × LD50 and brains processed from surviving mice at 1, 3, 7, and 14 days after paraoxon exposure (*n* = 3~5). (**B**) Representative IHC images of IBA1 staining in the hypothalamus, amygdala, and piriform cortex of mouse brain at 1 and 3 days after paraoxon exposures. The scale bars indicate 50 μm. (**C**) Quantitation of the IBA1-positive microglia in the hypothalamus, amygdala, and piriform cortex. The percent increase of IBA1+ cells is shown compared to the baseline level in naïve mice. Data are presented as mean ± SD. Statistical significance was compared between paraoxon-treated mice and naïve mice using one-way ANOVA. * *p* < 0.05, ** *p* < 0.01, and *** *p* < 0.001.

The LD50 for paraoxon in female BALB/c mice is routinely found to be 2.5 to 2.6 mg/kg [36]. All mice exposed to paraoxon at 0.85 × LD50 survive while experiencing severe cholinergic crisis (Racine symptom score = 6). To assess the effects of paraoxon-triggered cholinergic crisis on neuroinflammation, mice were exposed to paraoxon at 0.85 × LD50 or at 1 × LD50 as indicated. Microglial activation, a hallmark of neuroinflammation, was assessed using either molecular or immunohistochemical analyses for the expression of IBA1, a marker for microglial activation [26,28,39,40]. Analyses by RT-PCR of RNA isolated from brains at the indicated times after exposure revealed a ~2.6-fold increase in IBA1 mRNA at 9 h with the IBA1 mRNA levels apparently peaking with a ~3.7-fold increase at ~15 h after paraoxon (Figure 1A). At one day after exposure to paraoxon at 1 × LD50, immunohistochemical analyses of brains from the surviving mice showed a clear increase in cells staining for IBA1 protein and having the characteristic ameboid morphology of activated microglial cells [41,42]. This increase in IBA1+ microglia was seen in all three anatomical areas of the brain examined (hypothalamus, amygdala, and piriform cortex; see Figure 1B). The numbers of IBA1+ cells one day after exposure to paraoxon were increased by 57.3%, 40.9%, and 40.8% in the hypothalamus, amygdala, and piriform cortex, respectively (Figure 1C). Increases in IBA1+ microglial cells were also observed 3 days after exposures, although their numbers were somewhat reduced showing increases in IBA1+ cells over baseline of 21.6%, 29,6%, and 17.6% in the hypothalamus, amygdala, and piriform cortex, respectively. By 7 days and 14 days after paraoxon exposure, the numbers of activated microglia had returned to near-baseline levels. Collectively, these results demonstrate a rapid and robust induction of microglial activation in response to paraoxon exposures that last for days before returning to baseline.

**Figure 2:**
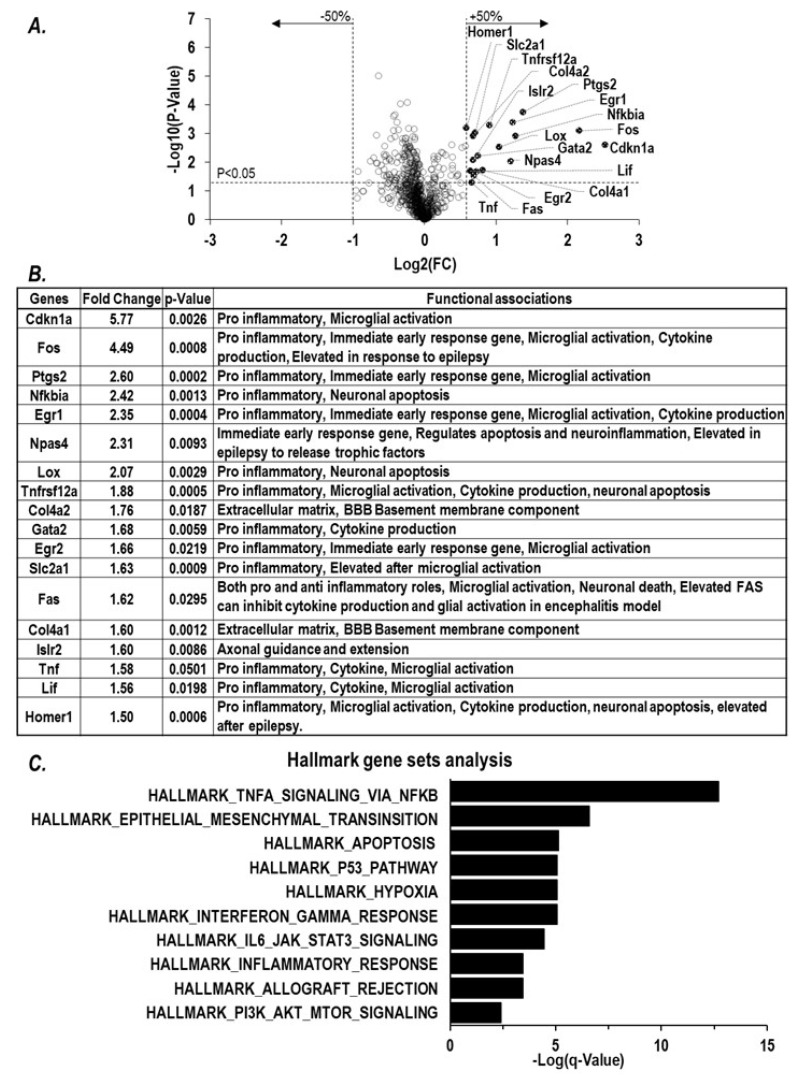
Paraoxon exposure induces neuroinflammatory transcriptional changes. Transcriptional changes were tested in the brains processed from surviving mice at 6 h after exposure to paraoxon at 1 × LD50 (*n* = 3). (**A**) Total RNA from brain lysates were analyzed using a NanoString neuropathology panel. Any genes with more than 50% change from the baseline from naïve mice and a *p*-value under 0.05 were considered to be differentially expressed genes (DEG). (**B**) Eighteen differentially expressed genes were identified, and their functional associations were explored. (**C**) GSEA mouse hallmark gene sets analysis was performed on the DEGs and the top 10 results listed.

We assessed transcriptomic changes triggered by paraoxon exposure using the NanoString nCounter neuropathology panel. Of 770 genes in this panel, 18 differentially expressed genes (DEGs) were found to be significantly upregulated in the brains of mice exposed to paraoxon 6 h earlier (Figure 2A). A literature-based search revealed that 15 of the 18 DEGs are known to be associated with inflammatory responses (Figure 2B).

We performed Gene Set Enrichment Analysis (GSEA) on the 18 DEGs and found functional relationships to the pathways of TNFα signaling, interferon, P53, IL6, KRAS, apoptosis, and inflammatory responses (Figure 2C). The gene most significantly upregulated in expression is Cdkn1a, which has been reported to be elevated in inflammation. [43,44]. The gene next most elevated in expression was *Fos*, an immediate early gene (IEG) associated with neuronal excitation and excitotoxity [45,46]. *Fos* is known to regulate the expression of several downstream inflammatory cytokines [47,48]. Interestingly, 5 of the 18 DEGs (*Fos*, *EGR1*, *EGR2*, *Npas4*, and *Ptgs2*) are classified as IEGs rapidly activated in response to various insults that are tied to microglial activation and to responses to oxidative stresses that can lead to neuronal cell death [49,50,51,52]. The genes Tnf and Lif were also found to be DEGs in our analyses. The expressions of these two cytokine genes have previously been reported to be elevated in the brains of mice exposed to organophosphates [53,54]. Taken together, our findings indicate that paraoxon exposures trigger neuroinflammatory signaling in the brain that involves a relatively rapid upregulation in the expression of genes associated with microglial activation, cytokine production, and neuronal apoptosis. Our analyses of gene expression in paraoxon-exposed mice thus reveal some details about the signaling pathways that connect OP exposures to brain damage.

### 2.2. Inflammatory Chemokine CCL2 Level Corelates with Extent of Cholinergic Symptoms

**Figure 3:**
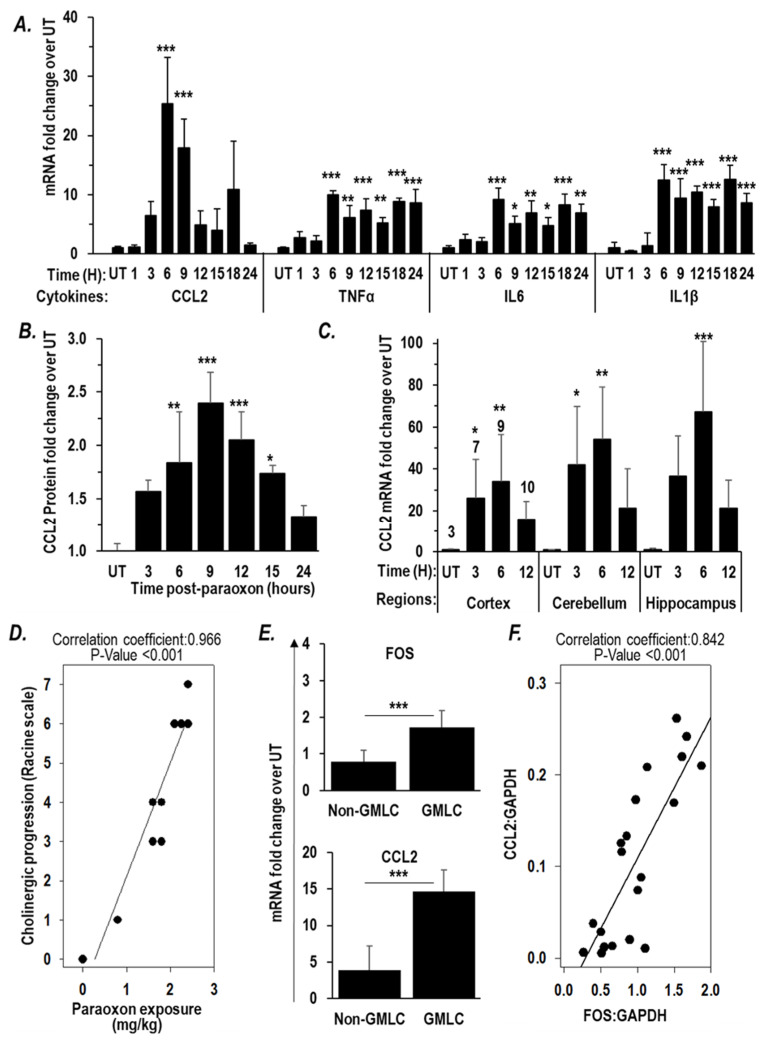
Paraoxon exposure increases inflammatory cytokine expression. (**A**,**B**) Mice were exposed to paraoxon at 0.85 × LD50 and brains were processed at the indicated times post-exposure (*n* = 3). (**A**) RT-PCR analysis of *TNF*α, *CCL2*, *IL6*, and *IL1β* mRNA levels in the whole brain. (**B**) ELISA assay for CCL2 protein level in the whole brain. (**C**) Mice were exposed to paraoxon at 1 × LD50 and brains of surviving mice were dissected into cortex, cerebellum, and hippocampus to perform RT-PCR analysis of *CCL2* mRNA levels at the indicated times. The numbers on each bar represents the numbers of mice represented by that data point. Data in (**A**–**C**) are presented as mean ± SD. Statistical significance was compared between paraoxon-treated mice and naïve mice using one-way ANOVA. * *p* < 0.05, ** *p* < 0.01, and *** *p* < 0.001. (**D**–**F**) Mice were exposed to varying doses of paraoxon at or under 1 × LD50 (*n* = 3/dose) and the cholinergic symptoms monitored at one hour after exposure using a modified Racine scale. Six hours after exposure, brains were removed and processed for RNA extraction. Shown are the correlation plots between (**D**) paraoxon exposure and Racine score, and (**E**) *Fos* and *CCL2* mRNA levels in mice that experienced grand-mal like seizures (GMLC; Racine = 6) and mice that did not (Non-GMLC), compared using a T-test. *** *p* < 0.001, and (**F**) *Fos* mRNA level and *CCL2* mRNA level using Pearson’s correlation method.

Since paraoxon can induce microglial activation and inflammatory signaling pathways via IEGs, we sought to examine the expression of those inflammatory cytokines known to be regulated by the two IEGs *Fos* and *EGR1* [47,48,49,50,55,56,57,58]. RT-PCR analyses of brain RNA isolated 6 h after paraoxon exposures revealed a significant increase in the mRNA levels of several inflammatory cytokines, namely, *TNF*α, *IL6*, *IL1β*, and *CCL2*. Of these genes, the increase in expression of *CCL2* was most robust increasing >25-fold by 6 h post-paraoxon (Figure 3A). ELISA assays confirmed an increase in CCL2 protein emanating from these elevated mRNA levels with maximal expression of the cytokine apparent at 9 h post-exposure (Figure 3B). We also assessed *CCL2* mRNA levels in dissected cortex, cerebellum, and hippocampus at various times after paraoxon exposure. As had been seen in the analyses of transcripts from the whole brain (Figure 3A), we saw significantly elevated *CCL2* mRNA expression in all three anatomical areas examined with a highest expression in each area seen 6 h after paraoxon exposure (Figure 3C). Because cholinergic pathways are found throughout the CNS [59], it is perhaps not surprising to find the neuroinflammatory responses triggered by a chemical with anticholinesterase activity to be correspondingly widespread.

We observed a very strong positive correlation (r = 0.966, *p* < 0.001) between paraoxon exposure levels and the intensity of cholinergic signs and symptoms (the Racine score that generally peaks during the first hour after exposure) (Figure 3D). Racine score 6 is assigned to mice that show grand mal-like seizures (GMLC). It is the highest point of escalation in Racine score before death. The mice that had GMLC showed a significantly higher *Fos* mRNA expression, 1.72-fold compared to the levels in naïve mice, while the mice that did not show grand mal-like seizure (non-GMLC) had 0.79-fold compared to the levels in naïve mice (Figure 3E). *Fos* is known to be a IEG that is upstream of CCL2 [48,55,56]. Accordingly, we found that the *CCL2* levels in the GMLC mice (14.6 -fold compared to the levels in naïve mice) were significantly higher than those in the non-GMLC mice (3.9-fold compared to the levels in naïve mice) (Figure 3E). Moreover, the *CCL2* mRNA levels correlated with *Fos* mRNA levels (r = 0.842, *p* < 0.001) (Figure 3F). Although it is not possible to define the relationships based only on these correlations, they are consistent with paraoxon-induced cholinergic crisis driving a rapid and robust surge in expression of *Fos* in response to excitotoxicity, and increased *Fos* expression in turn leads to an increase in expression *CCL2*, a representative of the inflammatory cytokines regulated by *Fos*. The observed upregulation of *CCL2* mRNA across all brain anatomical areas examined is consistent with neuroinflammation caused by cholinergic crisis affecting the various regions of the brain where cholinergic synapses are found.

### 2.3. scL-2PAM Is Very Effective in Reducing Neuroinflammation in the Brain

**Figure 4:**
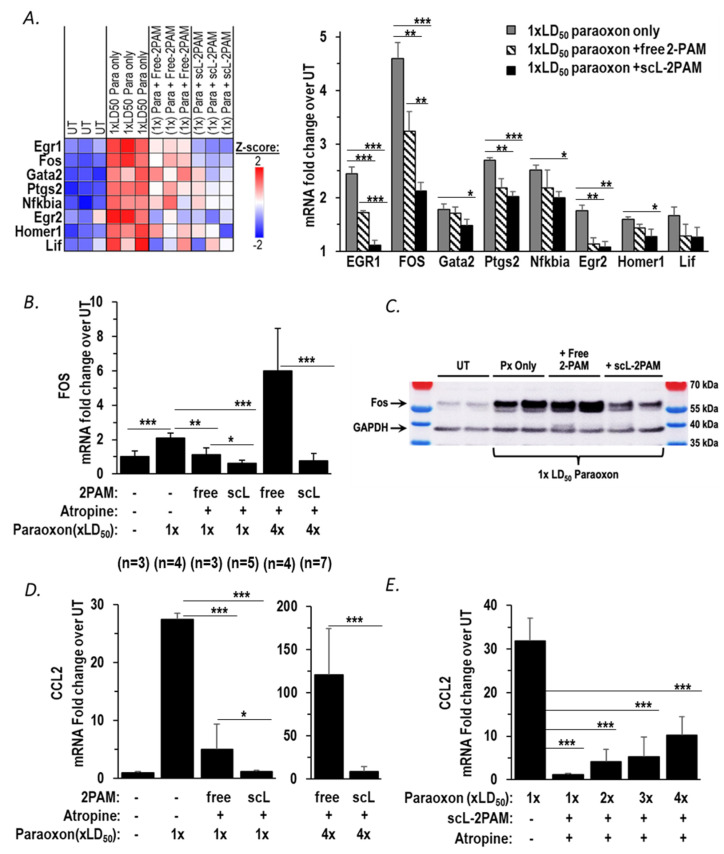
scL-2PAM mitigates the paraoxon-induced neuroinflammation and the surge in mRNA for the inflammatory cytokine CCL2. (**A**) NanoString neuropathology heatmap and quantitation of transcripts shown expressed as fold-changes in the indicated transcript levels in the brains of surviving mice 6 h after being exposed to paraoxon at 1 × LD50 or to paraoxon plus either scL-2PAM or free 2-PAM as monotherapies (*n* = 3/group). (**B**) *FOS* mRNA levels were quantitated by RT-PCR using RNA from brains of mice exposed to paraoxon (1 × LD50 or 4 × LD50 as indicated) and treated with either scL-2PAM or free 2-PAM plus atropine. The administration of the indicated antidote regimen was at 1 min after exposure to paraoxon, and brain RNA was isolated for analysis at 6 h. (**C**) c-Fos protein levels were assessed by western blot from brains of mice exposed to paraoxon at 1 × LD50 and treated with either scL-2PAM plus atropine or free 2-PAM plus atropine. The administration of the indicated antidote regimen was at 1 min after exposure to paraoxon, and brains were isolated for analysis at 1 h after paraoxon exposure. (**D**) *CCL2* mRNA expression was assessed in brains of surviving mice exposed to paraoxon at 1 × LD50 (*n* = 3) and treated with either scL-2PAM plus atropine (*n* = 6) or free 2-PAM plus atropine (*n* = 3). Groups of mice were also exposed to paraoxon at 4 × LD50 and treated with either scL-2PAM plus atropine (*n* = 7) or free 2-PAM plus atropine (*n* = 4). In all cases, *CCL2* mRNA levels in surviving mice were compared to those in naive mice (*n* = 3). Note the different scales for the fold-increase in *CCL2* expression depending upon the paraoxon exposure level. (**E**) scL-2PAM plus atropine was able to mitigate the increase in *CCL2* mRNA expression against escalating doses of paraoxon (1×, 2×, 3×, 4× LD50; *n* = 3–6/group). Data are presented as means ± SD, and statistical significance was determined using one-way ANOVA (* *p* ≤ 0.05, ** *p* ≤ 0.01, *** *p* ≤ 0.001).

We previously demonstrated that a novel nanocomplex encapsulating 2-PAM (termed scL-2PAM) is superior to unencapsulated 2-PAM in reactivating brain AChE activity, ameliorating cholinergic crisis, and rescuing mice from otherwise lethal exposures to paraoxon [36]. Aberrant or prolonged activation of neuroinflammatory pathways is a characteristic of OP-induced toxicity leading to seizures that can give way to status epilepticus. As a result, curbing neuroinflammation is now recognized as a therapeutic goal in countering OP exposures [24]. Based on our prior study of scL-2PAM, we hypothesized that scL-2PAM should be able to suppress the neurological sequelae of paraoxon exposure that is linked to neuroinflammation more effectively than the currently used 2-PAM formulation. Although 2-PAM is most often used in conjunction with atropine, we first examined the effect of scL-2PAM treatment as a monotherapy and assessed the expression of mRNAs associated with neuroinflammatory pathways. One minute after an exposure to paraoxon at 1 × LD50, mice were treated with either scL-2PAM or free 2-PAM. Six hours after paraoxon exposure, whole brains of surviving mice were processed for analyses of mRNA expression using NanoString neuropathology panels. Among the 18 DEGs that are elevated in response to paraoxon exposure (Figure 2B), 8 genes including *Fos* and *EGR1*, key regulators of inflammatory cytokine expression, were significantly reduced by scL-2PAM treatment as shown in the heatmap in Figure 4A. Although free 2-PAM treatment also reduced expression of some of these genes to a degree, the impact of free 2-PAM was modest compared to that seen with scL-2PAM (Figure 4A bar graphs). While Cdkn1a was significantly upregulated, our focus was on genes known to be directly involved in neuroinflammation. Future studies will explore the role of Cdkn1a in OP-induced neurotoxicity. These data indicate that the observed elevation of inflammatory gene expression seen after paraoxon exposures can be ameliorated by scL-2PAM, suggesting that it might reduce the neurological sequelae of paraoxon exposure that emanate from neuroinflammation.

The current standard therapy for OP intoxication consists of 2-PAM given in conjunction with a muscarinic ACh receptor antagonist (atropine) and a benzodiazepine anticonvulsant. Both the Mark I and DuoDote^®^ autoinjectors for first-line treatment of acute exposure to OPs contain 2-PAM and atropine as their active ingredients. Accordingly, we tested the ability of scL-2PAM to inhibit neuroinflammation when given in conjunction with atropine. We started by examining expression of *Fos*, an IEG driver of neuroinflammatory cytokine expression. Mice were exposed to paraoxon and then one minute later given either scL-2PAM or free 2-PAM at 25 mg/kg with atropine at 1.1 mg/kg. This dose of atropine was selected based on it being the approximate mouse equivalent of the atropine dose contained in three DuoDote^®^ autoinjectors, the number of autoinjectors recommended to be available for individuals at risk of OP exposure in the FDA-approved product label. *Fos* mRNA in the brains was assessed by RT-qPCR at 6 h after mice were exposed to paraoxon (Figure 4B). After an exposure to paraoxon at 1 × LD50 absent intervention, *Fos* mRNA levels in the brains of surviving mice were elevated 2.1-fold compared to the levels in naïve mice. Both scL-2PAM plus atropine or free 2-PAM plus atropine resulted in the abrogation of the *Fos* mRNA elevation with scL-2PAM being apparently more effective (Figure 4B). At a paraoxon exposure of 4 × LD50, there were no survivors absent intervention, but some animals were rescued by both forms of 2-PAM plus atropine. We previously reported that after exposures to paraoxon at 4 × LD50, the survival rate with scL-2PAM plus atropine was ~85%, whereas the survival rate with free 2-PAM plus atropine was only ~25% [36]. The superiority of scL-2PAM in abrogating the rise in *Fos* mRNA was clearly more apparent at 4 × LD50 paraoxon than had been seen at the lower exposure levels. The antidote regimen of scL-2PAM plus atropine resulted in *Fos* mRNA expression levels in surviving mice that were similar to those seen in brains of naïve mice, whereas in surviving mice receiving the combination of free 2-PAM plus atropine, *Fos* mRNA levels were elevated approximately sixfold above baseline levels (Figure 4B). To validate if the *Fos* mRNA expression pattern is also reflected at the protein level, we performed a Western blot using the brain tissue of mice exposed to paraoxon at 1 × LD50 for 1 h (Figure 4C). This early time point was chosen for the protein analysis based on c-Fos being established as an immediate early gene (IEG) in other organophosphate studies [38,60] and its short protein half-life of approximately 2 h [46,61]. The Western blot results corroborate our mRNA findings. Untreated (UT) mice showed a relatively light band of c-Fos, indicative of basal levels. Mice that were exposed to paraoxon only (i.e., absent any antidotes) showed a strong c-Fos band reflecting higher levels of c-Fos in the brains of these mice (Figure 4C). Treatment with free 2-PAM plus atropine did not appreciably reduce c-Fos levels. In contrast, mice exposed to paraoxon and treated with scL-2PAM plus atropine displayed substantially reduced levels of c-Fos protein. It is apparent from these results that the antidote regimen based on scL-2PAM is significantly more capable than free-2PAM treatment in curbing the paraoxon-triggered c-Fos induction seen at both the transcript and protein levels.

Based on the impact of antidote regimens on *Fos* expression and our prior observation of a positive correlation between *Fos* mRNA levels and *CCL2* mRNA levels, we compared the ability of scL-2PAM plus atropine with that of free 2-PAM plus atropine to reduce expression of this inflammatory cytokine. When mice were exposed to paraoxon at 1 × LD50 absent antidotes, *CCL2* expression was elevated >27-fold in the brains of surviving mice. In mice exposed to paraoxon at this level, both scL-2PAM plus atropine and free 2-PAM plus atropine resulted in significantly lower expression of *CCL2* mRNA. In the case of the regimen based on scL-2PAM, *CCL2* mRNA was only ~1.2-fold above baseline, whereas with the regimen based on free 2-PAM, *CCL2* mRNA levels were still ~5-fold above baseline expression (Figure 4D, left panel). With paraoxon exposure increased to 4 × LD50, brain *CCL2* mRNA levels were elevated >120-fold in surviving mice receiving the free-2PAM-based regimen suggestive of robust neuroinflammation in these animals. In contrast, in surviving mice receiving scL-2PAM plus atropine, *CCL2* mRNA was much more modestly elevated (approximately eightfold over baseline). In aggregate, these data confirm that *CCL2* mRNA levels are markedly elevated in a manner dependent on the levels of paraoxon exposure and that the expression of this inflammatory cytokine can be reduced more effectively by an antidote regimen based on scL-2PAM than by one based on free 2-PAM.

To assess if the expression of genes exhibits a dose–response curve with regard to OP exposures, we compared the *CCL2* mRNA levels in the brains of mice exposed to progressively escalating levels of paraoxon (between 1 × LD50 to 4 × LD50) that had been rescued by receiving scL-2PAM plus atropine. There were no survivors at the higher paraoxon levels absent intervention. Above 1 × LD50, paraoxon kills 100% of the mice absent intervention, as noted earlier. As shown in Figure 4E, levels of *CCL2* mRNA were >30-fold increased upon exposure to paraoxon at 1 × LD50 (comparable to that seen in Figure 4D). After receiving scL-2PAM plus atropine, brain *CCL2* mRNA levels in the mice exposed to paraoxon at 1 × LD50 were only increased ~1.2-fold from the baseline level in naïve mice. As paraoxon exposures increased, there was a progressive increase in *CCL2* mRNA in the brains of the mice that had been rescued with scL-2PAM plus atropine. However, even with a paraoxon exposure as high as 4 × LD50, *CCL2* mRNA levels were lower than those in mice exposed only to paraoxon at 1 × LD50. It is evident from these data that escalating paraoxon exposures causes a corresponding increase in *CCL2* mRNA expression, but scL-2PAM plus atropine can clearly blunt the rise in the expression of *CCL2* mRNA. These findings suggest that this treatment regimen has the potential to reduce OP-triggered neuroinflammation and ameliorate the longer-term sequelae of OP exposure.

### 2.4. scL-2PAM Treatment Curbs the Paraoxon-Driven Microglial Activation

**Figure 5:**
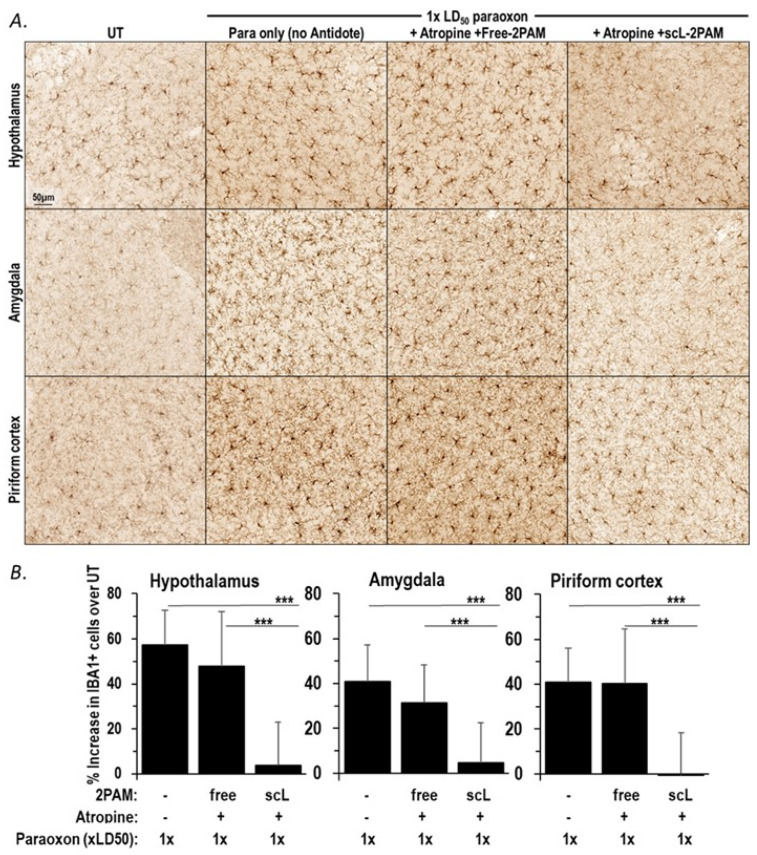
scL-2PAM treatment curbs the paraoxon driven microglial activation. Mice were exposed to paraoxon at 1 × LD50 and then treated with either scL-2PAM plus atropine or free 2-PAM plus atropine 1 min later. Brains were processed 1 day later for immunohistochemical evaluation of the numbers of activated microglia (IBA1+ cells). (**A**) Representative images of IBA1+ cells in the hypothalamus, amygdala, and piriform cortex from surviving mice are shown. (**B**) Quantification of the increase in the number of IBA1+ cells detected by IBA1 IHC (*n* = 3–7/group) when compared to the number of IBA1+ cells in comparable areas of the brains of naive mice. Data are presented as means ± SD, and statistical significance was determined using one-way ANOVA (*** *p* ≤ 0.001).

OP exposures trigger microglial activation with an increase in inflammatory cytokines, and this neuroinflammatory pathway plays a crucial role in causing longer-term neuropathogenesis [24,29]. When mice were exposed to paraoxon, we observed an early rise in cytokine expression indicative of microglial activation (see Figure 1B and Figure 3A). The levels of cytokine mRNA expression were reduced after treatment with scL-2PAM suggesting its potential to block the brain damage emanating from OP exposures and consequent neuroinflammation. To directly assess, the impact of scL-2PAM based treatments on glial cell activation, mice exposed to paraoxon with and without antidotes comprising either scL-2PAM plus atropine or free 2-PAM plus atropine were examined using IBA1 immunohistochemistry. At 24 h exposure to paraoxon at 1×LD50, brains were assessed for their microglial status. As described above, the number of IBA1+ cells were significantly elevated after paraoxon exposures, and the cells displayed the classic ameboid morphology of activated microglia. This visible increase in the number of IBA1+ cells was observed in the hypothalamus, piriform cortex, and amygdala. When scL-2PAM plus atropine was given as the antidote regimen, the numbers of IBA1+ cells were found to be very similar to those seen in mice never exposed to paraoxon (Figure 5). Moreover, the microglia in these mice appeared to be largely ramified, indicating that this treatment regimen had reduced the microglial activation triggered by paraoxon. In contrast, microglial activation was not appreciably prevented by a treatment regimen involving free 2-PAM plus atropine. This pattern of scL-2PAM superiority over free 2-PAM was seen in all three of the brain anatomical areas examined (hypothalamus, piriform cortex, and amygdala). Quantitation of the IBA1+ cells revealed a significantly lower numbers of activated microglia after scL-2PAM plus atropine treatment in all three brain areas (Figure 5B). Paraoxon exposures resulted in 57.3%, 40.8%, and 40.9% increases in the number of IBA1+ cells in the hypothalamus, piriform cortex, and amygdala, respectively. Free 2-PAM plus atropine treatment was not effective at reducing these elevated numbers of IBA1+ cells, whereas mice receiving scL-2PAM plus atropine displayed near-baseline numbers of IBA1+ cells in all three anatomical areas. These immunohistochemical results are consistent with our analysis of transcript levels that indicated that compared to free 2-PAM, scL-2PAM is better at curbing the neuroinflammation arising from exposures to paraoxon.

### 2.5. scL-2PAM Treatment Ameliorates Brain Damage after Paraoxon Exposure

**Figure 6:**
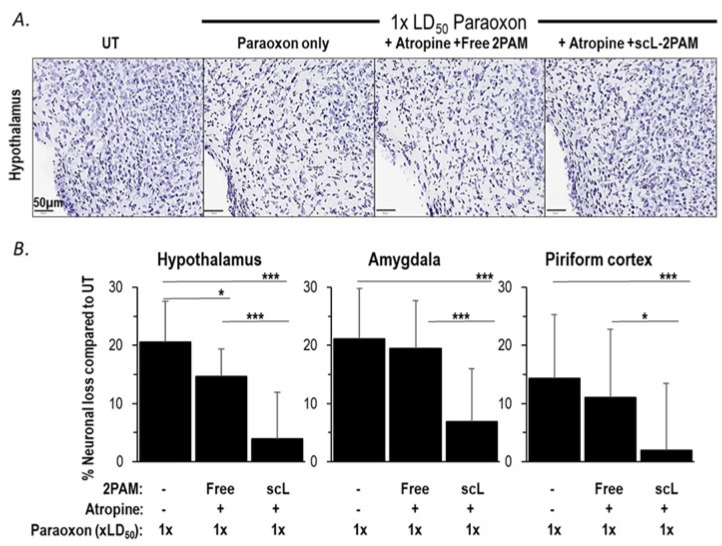
scL-2PAM treatment ameliorates paraoxon induced neuronal loss. Mice were exposed as indicated to paraoxon at 1 × LD50 or 4 × LD50 and 1 min later administered either free 2-PAM plus atropine or scL-2PAM plus atropine. Brains were processed at 14 days for quantitation of healthy neurons (Nissl+ cells). (**A**) Representative images of Nissl+ cells in the hypothalamus. (**B**) Percentage loss in Nissl+ cells 14 days after exposure to only paraoxon at 1 × LD50 or with the indicated treatments. Data are expressed as the percentage of neurons lost compared to the numbers seen in the brains of naive mice (*n* = 6–9/group). Shown are the loss of neurons in the hypothalamus, amygdala, and piriform cortex. Data are presented as mean ± SD, and statistical significance was determined using one-way ANOVA (* *p* ≤ 0.05, *** *p* ≤ 0.001).

OPs cause persistent neuropathology by pushing brain cells into apoptotic death [62]. Ultimately, the longer-term sequelae of OP exposures that matters most is the irreversible death of brain cells. Based on the ability of scL-2PAM plus atropine to suppress paraoxon-triggered microglial activation and cytokine mRNA expression, we sought to assess the ability of this treatment regimen to prevent brain damage using Nissl staining that reveals healthy neurons in the brain. Fourteen days after paraoxon exposures, significant losses of neurons were observed in the hypothalamus, piriform cortex, and amygdala (Figure 6). Treatments with free 2-PAM plus atropine were largely ineffective in preventing these neuronal losses. However, treatments involving scL-2PAM plus atropine were able to reduce significantly the neuronal losses in the hypothalamus (only 3.9% neuronal loss compared to 20.6% loss compared to 21.1%), the piriform cortex (only 2.0% neuronal loss compared to 14.4% loss), and the amygdala (only 6.9% neuronal loss). Collectively, these findings demonstrate that scL-2PAM plus atropine can protect against the brain damage caused by paraoxon exposures by significantly reducing neuronal cell losses in the brain. The antidote regimen involving free 2-PAM is relatively ineffective in preventing brain damage.

**Figure 7:**
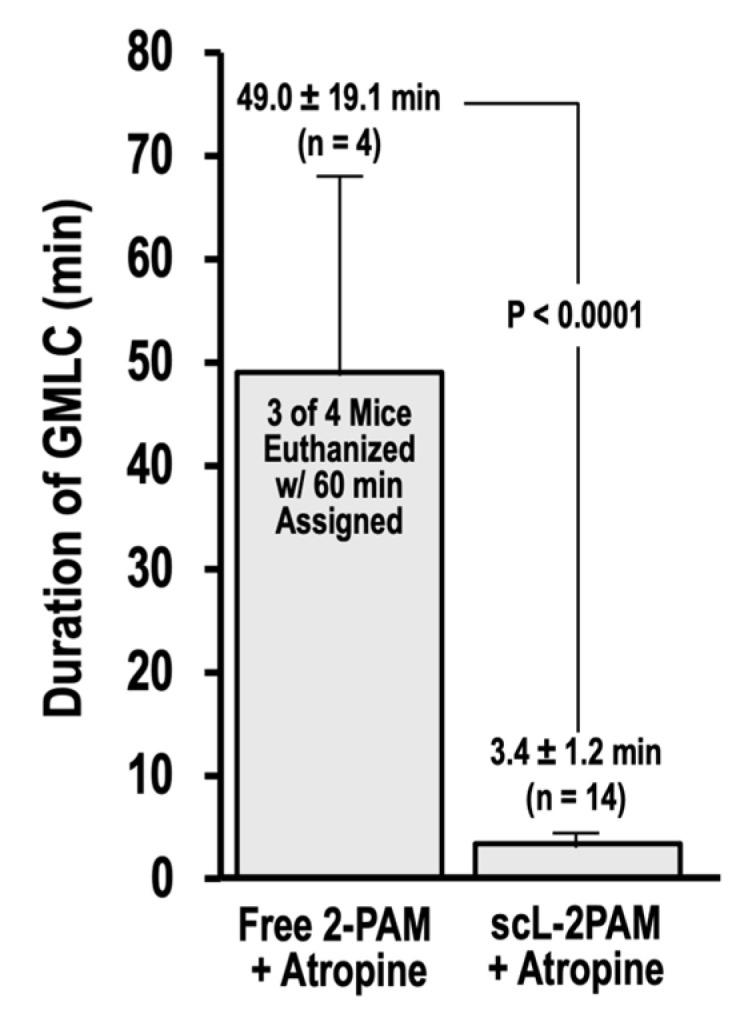
scL-2PAM treatment reduces the time spent in grand mal-like convulsions. Mice were exposed as indicated to paraoxon at 4 × LD50 and 1 min later administered either free 2-PAM plus atropine or scL-2PAM plus atropine (oxime at 25 mg/kg and atropine at 1.1 mg/kg). Mice were monitored continuously, and cholinergic signs and symptoms noted. Shown is the time spent in grand mal-like convulsions (GMLC; Racine = 6) under respective treatments. When GMLC duration reached 60 min, the mice were euthanized and a duration time of 60 min assigned. Three of four surviving mice that had received the antidote regimen based on free 2-PAM were euthanized. None of the 14 survivors receiving the antidote regimen based on scL-2PAM were euthanized. *p* values for time from paraoxon to the end of GMLC were determined by t tests.

Exposures to OPs with consequent ACh excitotoxity can lead to seizures including SE [63]. SE is a serious complication that is defined as a seizure that lasts more than five minutes without regaining consciousness [64]. There is considerable evidence that SE causes neuropathology [65,66,67]. SE represents a complex challenge in treatment of OP toxicity because of its association with long-term cognitive and behavioral deficits [68,69]. Accordingly, we sought to compare antidote regimens based on either free 2-PAM or scL-2PAM for their ability to curb seizures triggered by paraoxon exposures (Figure 7). In this experiment, mice were given the antidotes 1 min after paraoxon and were continuously monitored, with the timing of cholinergic signs and symptoms noted. A Racine score of 6 is assigned to severe convulsions that resemble grand mal-like seizures. An important factor in terms of causing neuropathology (loss of neurons) is the duration of seizures. We found that three of the four survivors in the free 2-PAM group continued in GMLC for 60 min when they were humanely euthanized. In stark contrast, survivors in the scL-2PAM group exited GMLC in under 4 min on average, and no animals required euthanization. The total time from paraoxon to the end of GMLC in this group was only 3.4 ± 1.2 min compared to 49 ± 19.1 min in the free 2-PAM group.

## 3. Discussion

A broadly recognized framework for understanding OP toxicities is based on the primary mechanism of action of these chemical agents, i.e., inhibition of AChE [70]. The inactivation of AchE results in Ach accumulation in the synapses that leads to excitotoxity, culminating in cholinergic crisis with seizures that can produce SE and brain damage. With lethal exposures to OPs, death due to respiratory failure appears to involve inhibition of the brain’s respiratory control center [9,71]. At sub-lethal levels, OPs can still result in onset of neuroinflammation that is characterized by microglial activation resulting in a cytokine surge and irreversible brain damage with long-term neurological sequelae in survivors [17,26,27,28]. The development seizures from Ach-driven excitotoxicity is considered to be a key driver of neurological consequences that result from OP exposures. [22,23]. In our study, we observed that in the brains of mice surviving paraoxon exposures elevated expression of several IEGs, including *Fos*, *EGR1*, *EGR2*, and *Ptgs2*, and *Npas4* was also observed. Elevated expressions of these genes have been implicated in early responses to a variety of insults to the brain [45,47,49,51,52,72]. In particular, elevation of *Fos* mRNA has been linked to excitotoxicity [45,46,73] and to upregulation of downstream participants in neuroinflammation (i.e., inflammatory cytokines). In our study, *Fos* expression was increased as OP-triggered cholinergic crisis intensified, consistent with the observations of others [38,60,74]. *Fos* and *Egr1* are known to upregulate the inflammatory cytokines, including *CCL2*, *TNF*α, *IL1β*, and *IL6* [49,55,56], and the levels of transcripts encoding these cytokines were also elevated by paraoxon exposures in our mice. Levels of *CCL2* mRNA were found to be the most dramatically elevated in the cytokines assessed, and *CCL2* mRNA levels displayed a strong correlation with *Fos* mRNA levels. A self-propagating cytokine surge (sometimes called a cytokine storm) is the hallmark neuroinflammatory pathway in which microglial activation participates [25]. The most widely used marker for activation of microglia is expression of IBA1, and an increase in this marker protein has been observed as a consequence of organophosphate exposures. [17,26,27,28]. Consistent with these findings, we observed a relatively early surge in IBA1 mRNA expression, and IBA1 immunohistochemistry revealed microglial activation in the hypothalamus, amygdala, and piriform cortex that peaked at one day after paraoxon exposures and persisted for up to three days. In short, all of our observations align with the notion that OPs trigger a rapid and prolonged neuroinflammatory response in multiple anatomical regions of the brain.

However, it has also been recognized for some time that a limitation of 2-PAM relates to the fact that it inefficiently crosses the BBB to reactivate inactivated AchE in the brain [34,35]. Kuruba et al. and Putra et al. found that 2-PAM given in conjunction with atropine was not able to mitigate the microglial activation and neuronal damage caused by DFP exposure in rats [2,20]. Similarly, Finkelstein et al. found that 2-PAM treatment was not able to rescue from the neurological consequences of paraoxon exposure in rats [17]. A similar lack of efficacy of 2-PAM in protecting from OP-driven neuroinflammation and neuronal damage has been observed by several other groups [27,28,33,75]. In our study, we conducted head-to-head comparisons of unencapsulated 2-PAM (termed “free 2-PAM” here) with a novel nanocomplex formulation of 2-PAM (scL-2PAM) that was designed to take the oxime AchE reactivator across the BBB. We examined the ability of free-2PAM to curb microglial activation and the accompanying expression of neuroinflammatory genes after paraoxon exposures. We used the surge in IEG expression as well as microglial activation as indicators to demonstrate the effects of OP exposure and their mitigation by scL-2PAM treatment in a way that results in reduced brain damage. We did not study nor suggest a direct interaction between scL-2PAM and microglia or of any direct effect of scL-2PAM on IEG expression levels. In our study, free-2PAM was able to mitigate the increase in *Fos* and *CCL2* expression caused by paraoxon at 1 × LD50 to some extent but was not very effective when paraoxon exposures were raised to 4 × LD50. These results are in line with our previous report showing that giving mice free-2PAM plus atropine provided some improvement in survival of paraoxon-exposed mice [36].

We previously published a study that demonstrated that scL-2PAM designed to cross the BBB is superior to free 2-PAM in terms of ability to reactivate OP-inactivated AchE in the brains of OP-exposed mice [36]. This study also documented the superiority of scL-2PAM in curtailing the severity and duration of cholinergic crisis and in rescuing animals from otherwise-lethal exposures of paraoxon. Since AchE activity and cholinergic crisis are key drivers of neurological damage, we sought to evaluate in this follow-on study the ability of scL-2PAM to ameliorate the neuroinflammatory consequences of paraoxon exposure and to prevent longer-term brain damage in survivors of sub-lethal paraoxon exposures. Mice that survived exposures to paraoxon at 1 × LD50 received antidote regimens based on either free 2-PAM or scL-2PAM. Mice receiving scL-2PAM were found to have lower levels of *Fos* and *CCL2* mRNAs in their brains when compared to mice receiving free-2PAM instead. Even mice that survived paraoxon at 4 × LD50 as a result of the antidotes had lower levels of *Fos* and *CCL2* transcripts in their brains if they received the antidote regimen based on scL-2PAM.

In addition to its impact on our transcriptomic analyses, the scL-2PAM-based treatment regimen was able to mitigate microglial activation in the hypothalamus, amygdala, and piriform cortex following paraoxon, as evidenced by IBA1 immunohistochemistry. The treatment regimen that included free 2-PAM was incapable of blocking microglial activation. These findings clearly demonstrate that scL-2PAM can overcome the limitations of free 2-PAM as a countermeasure against OP-triggered neuroinflammation. It has been previously reported that paraoxon exposure causes blatant neuronal damage in hippocampus, thalamus, piriform cortex, and amygdala [17,76,77] Other groups have also reported paraoxon exposure leading to neuronal damage in survivors [78,79,80,81]. The ideal countermeasure would be one that is capable of preventing neuronal cell death after OP exposure. In the current study, we confirmed that paraoxon causes neuronal loss in the hypothalamus, piriform cortex, and amygdala that is seen on day 14 after exposures. This neuronal loss was observed in the same regions where persistent microglial activation was observed, supporting the notion that OPs cause neuroinflammation in survivors, which culminates in losses of neurons. We found that antidote regiments based on scL-2PAM were able to reduce neuronal losses in three regions of the brain and that this prevention of brain damage was not seen in mice given free 2-PAM instead of scL-2PAM. Given that prolonged seizures cause neuropathology [65,66,67], the basis for the amelioration of neuronal losses by antidote regimens based on scL-2PAM is likely linked to the ability of this nanoformulation of the AchE reactivator to dramatically curtail the time spent in GMLC after paraoxon exposures (Figure 7). Our findings support the notion that the mechanism of action in scL-2PAM is AChE reactivation, leading to reduced acetylcholine accumulation and less excitotoxicity. Consequently, we did not assess any direct effects of scL-2PAM on microglia or expression of immediate early genes (IEGs) in this study, as all our data indicate that the observed neuroprotective benefits stem from its role in counteracting AChE inhibition by organophosphates. Overall, paraoxon exposure-driven cholinergic crisis lead to neuroinflammatory consequences culminating in neuronal loss. These consequences were substantially curbed by scL-2PAM treatment as compared to the currently used free 2-PAM treatment.

## 4. Materials and Methods

### 4.1. Chemicals

Cationic lipid 1,2-dioleoyl-3-trimethylammonium propane (DOTAP) and neutral lipid dioleolylphosphatidyl ethanolamine (DOPE) were purchased from Avanti Polar Lipids (Alabaster, AL, USA). Paraoxon (Diethyl p-nitrophenyl phosphate) was purchased from Santa Cruz Biotechnology (Dallas, TX, USA). Atropine sulfate (1 mg/mL) and pralidoxime (2-PAM) were purchased from American Regent (Shirley, NY, USA) and TCI Chemicals (Tokyo, Japan), respectively. Heparin (1000 USP/mL) was purchased from Fresenius Kabi (Lake Zurich, IL, USA). Paraformaldehyde (16% solution) was purchased from Electron Microscopy Sciences (Hatfield, PA, USA). Sucrose was obtained from Sigma (St. Louis, MO, USA) and MP Biomedicals (Santa Ana, CA, USA). Sterile endotoxin-free LAL reagent water was purchased from Lonza (Walkersville, MD, USA).

### 4.2. Paraoxon Preparation

Paraoxon is an oily liquid. To obtain the working solutions of paraoxon used in the experiments, the commercially available paraoxon (1.27 g/mL) was serially diluted in ordinary extra virgin olive oil. Paraoxon and oil were mixed by vortexing at high speed for one minute and rotated at 10 rpm at room temperature using the Benchmark RotoBot™ Programmable Rotator (Benchmark Scientific, Sayreville, NJ, USA). The working stock solutions were 1.5 or 0.25 mg/mL, and these were used to expose mice to paraoxon at the desired level.

### 4.3. Preparation of scL Nanocomplex Encapsulating 2-PAM

A nanocomplex encapsulating 2-PAM (scL-2PAM) was prepared as previously described [36]. Briefly, 2-PAM was incorporated into the liposomes comprising a 1:1 mixture of DOTAP and DOPE at the molar ratio of lipids/2-PAM of 1:8 by the ethanol injection method. The full scL-2PAM nanocomplex was prepared by simple mixing by inversion of the targeting moiety [recombinant anti-transferrin receptor single chain antibody (TfRscFv)] and liposomes encapsulating 2-PAM (Lip-2PAM). Particle size, polydispersity index (a measure of the homogeneity of the population), and zeta potential (a measure of the charge of the nanocomplex) of scL-2PAM nanocomplexes were determined by dynamic light scattering at room temperature using the Malvern Zetasizer Nano ZS (Worcestershire, UK). For the mouse injections, 5% sucrose (final concentration) as an excipient was used in injections of both free 2-PAM and scL-2PAM. After the preparation of scL-2PAM (in 5% sucrose), samples were flash-frozen in liquid nitrogen and lyophilized to dryness (three to four days) using a Labconco Freeze Dryer System (Kansas City, MO, USA). Upon removal from the lyophilizer, the tubes were sealed with parafilm and stored with desiccant at 4 °C. To reconstitute the lyophilized scL-2PAM, sterile, endotoxin-free LAL water equivalent to the original volume was added to the lyophilized scL-2PAM (yielding 2.08 mg 2-PAM/mL in 5% sucrose). The tube was inverted 10 times to solvate the lyophilized material, and then rotated by inversion at 30 rpm for 10 min at room temperature on the rotator. Prior to injection and based on the weight of individual mice, the reconstituted scL-2PAM was diluted with sterile, endotoxin-free 5% sucrose to yield the required dose in an injection volume of 0.25 mL. Free 2-PAM was dissolved at a final concentration of 100 mM in sterile, endotoxin-free 5% sucrose. Prior to injection and based on the weight of individual mice, the stock 2-PAM solution was diluted in 5% sucrose to yield the desired dose in an injection volume of 0.25 mL.

### 4.4. Animal Studies

All animal experiments were approved by the Georgetown University Institutional Animal Care and Use Committee and by the Animal Care & Use Review Office (ACURO) of the U.S. Department of Defense. All experiments were performed in the AAALAC-accredited, USDA-registered animal facility of Georgetown University abiding by the Guide for the Care and Use of Laboratory Animals as mandated by the Office of Laboratory Animal Welfare (OLAW). Six-week-old, female BALB/c AnNHsd mice (Harlan/Envigo, Indianapolis, IN, USA) were housed five per cage with corncob bedding at 20–22 °C in a 12 h light/dark cycle with 30–70% relative humidity and received food and water ad libitum. Mice were quarantined upon arrival and allowed to acclimate to their cage environment for at least 3 days before use. The LD50 for paraoxon (the dose that yields 50% survival) was established by injecting intraperitoneally (IP) at least 4 mice/dose with increasing doses of paraoxon. The LD50 was generally found to fall between 2.5 to 2.75 mg/kg. For studies assessing the ability of free 2-PAM or scL-2PAM to suppress neuroinflammation, the mice were injected IP on the right side with the paraoxon at the indicated dose. Approximately one minute after paraoxon administration, mice were injected IP on the upper left side with 25 mg/kg scL-2PAM or free 2-PAM. If atropine was included, the atropine was injected IP immediately after 2-PAM on the lower left side. Based upon the weight of individual mice, atropine (1 mg/mL) was diluted with sterile, endotoxin-free 5% sucrose solution to achieve a dose of 1.1 mg/kg in an injection volume of 0.15 mL.

### 4.5. Assessment of Cholinergic Crisis with Racine Scale

The intensity of cholinergic crisis induced by paraoxon exposure was assessed as previously described [36]. In brief, mouse behavior was monitored continuously for one hour after the exposure to varying doses of paraoxon for symptoms associated with cholinergic crisis. These behaviors were scored using modified Racine scale [21,40,82] with score 0 = normal behavior (walking, bright and alert movements); 1 = lethargic movements, startled, immobile; 2 = mild tremors, head bobbing; 3 = tremors and twitching, extensions of body muscles; 4 = early seizure-like symptoms, partial body clonus; 5 = sustained seizure-like symptoms, loss of posture, myoclonic jerks, behavior consistent with status epilepticus; 6 = intense seizure-like symptoms, repetitive jumping or bouncing, wild running; and 7 = death.

### 4.6. Tissue Collection for Gene Expression Analyses

Mice brains were removed immediately upon death or at indicated times after paraoxon exposure. The whole brains were rinsed in 1xPBS, minced into small pieces, and flash frozen in liquid nitrogen. In some experiments, cortex, hippocampus, and cerebellum were dissected before being flash frozen. The frozen tissues were pulverized with Bessman Tissue pulverizer (Fisher Scientific, Waltham, MA, USA) into powdered form and stored at −80 °C.

### 4.7. Quantitative RT-PCR

About 30 mg of pulverized brain tissue was lysed and homogenized using a tissue homogenizer. Tissue Master 125 (OMNI international, Kennesaw, GA, USA) to extract total RNA using Pure Link RNA Mini Kit (Life technologies, Carlsbad, CA, USA) according to the manufacturer’s instructions. RNA concentration and quality were determined using ND-1000 NanoDrop spectrophotometer (Thermo scientific, Waltham, MA, USA). A total of 500 ng of RNA was converted into cDNA using iScript cDNA synthesis kit (Bio-Rad, Hercules, CA, USA). Quantitative RT-PCR (RT-qPCR) was performed using 100 ng cDNA template, TaqMan Fast Advanced master mix (Life technology), and TaqMan assays for mouse IBA1 (MM00479862_g1), FOS (MM01253033_m1), *CCL2* (Mm00441242_m1), *TNF*α (qMmuCEP0028054), *IL6* (qMmuCEP0054186), and *IL1β* (qMmuCEP0054181) on a Quant Studio 3 instrument (Life Technologies). Data were analyzed using the ∆∆Ct method and normalized to housekeeping gene mouse GAPDH (Mm99999915_g1). The fold-change in mRNA expression compared to untreated (UT) brains was calculated.

### 4.8. NanoString nCounter Gene Expression Analysis

To investigate the changes in gene expression pattern in the brains of mice that were exposed to paraoxon with or without antidotes, the Neuropathology panel of nCounter Gene Expression Assay (NanoString Technologies, Seattle, WA, USA) was used. After testing for the integrity of RNA samples using Agilent Bioanalyzer (Santa Clara, CA, USA), 500 ng of total RNA was used as input for the nCounter analysis performed by Genomics and Epigenomics Shared Resource at the Georgetown Lombardi Comprehensive Cancer Center. Raw data were normalized to the housekeeping gene expressions using nSolver 4.0 (NanoString Technologies). Genes that had normalized counts above 20 were considered for the subsequent analyses. Fold-change in expression compared to baseline values for each gene was calculated and compared to identify differentially expressed genes (DEGs) in response to paraoxon exposure, The selection criteria were an increase of at least 1.5-fold from baseline and *p* < 0.05. Morpheus web-based software (https://software.broadinstitute.org/morpheus, accessed on 1 September 2022) was used to generate the heatmaps of gene expression. Gene set enrichment analysis was performed to identify biological pathways that are associated with the DEGs observed after paraoxon exposure. MH: hallmark gene sets from Mouse Molecular Signatures Database (MSigDB, (http://www.broadinstitute.org/gsea/msigdb), accessed on 1 September 2022) were used for the analysis. The significance threshold for pathway enrichment was set at 0.05 FDR (q-value). Results of the top 10 pathways recognized were displayed in the results.

### 4.9. CCL2 Protein ELISA

Approximately 50 µg of pulverized frozen brain tissue was lysed in radioimmunoprecipitation assay (RIPA) lysis buffer (1% NP40, 0.5% sodium deoxycholate, 0.1% SDS) with inhibitors aprotinin, phenylmethanesulfonyl fluoride, and orthovanadate (Sigma, St. Louis, MO, USA). The protein concentration of the lysates was measured using Pierce BCA Protein Assay kit (Thermo Fisher, Waltham, MA, USA). The CCL2 in the brain lysates were measured using the mouse CCL2 DuoSet ELISA kit (R&D Systems, Minneapolis, MN, USA). The CCL2 levels were normalized to each samples total protein and the fold-changes relative to baseline values were calculated.

### 4.10. Western Blot

Equal amounts of protein (30 µg) from each sample were resolved by NuPAGE™ Bis-Tris Midi Protein Gels, 4 to 12% Invitrogen, Waltham, MA, USA). Primary antibody against c-Fos (ABCAM, Cambridge, United Kingdom) to evaluate Fos expression and antibody against GAPDH (R&D Systems, Minneapolis, MN, USA) were used to as an internal control to assess gel loading. Membranes were incubated with secondary antibodies from Jackson ImmunoResearch Labs (West Grove, PA, USA) and Thermo Scientific (Waltham, MA, USA). Bands were detected using detection substrate (Azure Biosystems, Dublin, CA, USA) and imaged with the Amersham Imager 600 (GE, Boston, MA, USA).

### 4.11. Histology

Mice were sedated with ketamine/xylazine cocktail and perfused by exposing the heart with thoracotomy and inserting a needle into the left ventricle to flush with 10 mL PBS containing heparin (10 units/mL). PBS was switched with 50 mL of 4% paraformaldehyde (PFA) solution to continue the perfusion on a pump at a flow rate of 3.3 mL/min to pre-fix the brain. Collected brains were post-fixed in 4% PFA for at least 6 h, switched into 20% sucrose in 0.1 M PBS buffer (pH 7.4) for cryoprotection for 72 h, and then snap frozen in pre-cooled isopentane with dry ice. Frozen brains were stored at −80 °C until the histological analyses. Mouse brains were cryo-sectioned into 40 μm coronal sections and three successive sections containing cortex and hippocampus (approximately −1.22~−2.46 mm from the bregma) from each brain were processed for either immunohistochemistry for Iba1 or Nissl stain using FD cresyl violet solutionTM (FD NeuroTechnologies, Inc., Columbia, MD, USA). The slides were scanned using APERIO GT450 (Leica biosystems, Wetzlar, Germany) and digitized images were processed for the quantification for either IBA1+ or Nissl+ cells using QuPath 2.3. [83]. Three regions of interest (ROIs), 62,500 μm^2^/ROI were counted per sample for each brain anatomical area examined. For both IBA1 and Nissl stain, average cell counts from untreated brains were used to calculate the percentage change in cell numbers in the brains from mice in the various experimental groups. For IBA1, percentage increase in IBA1+ cells over baseline was calculated, while for Nissl, percentage of neurons lost compared to baseline was calculated.

### 4.12. Statistics

All the data shown are represented as mean ± standard deviation (SD). The statistical comparison to the relative control values were performed using the one-way ANOVA test for multiple groups or t-test for comparison between 2 groups using Sigma plot version 11.0 (Systat Software, Chicago, IL, USA).

## 5. Conclusions

Collectively, our results suggest that if OP-triggered activation of microglia and neuroinflammation via IEGs and consequent neuronal damage could be curbed effectively, it might be possible to mitigate the longer-term neurocognitive disorders observed in human survivors after OP exposure. Our data indicate that scL-2PAM, a novel formulation of the AchE reactivator designed to cross the BBB, can indeed ameliorate paraoxon-triggered neuroinflammation and mitigate against paraoxon-caused brain damage. This study, along with our previous article [36], demonstrates that scL-2PAM acts via improved AchE reactivation in the CNS, the curbing of cholinergic crisis symptoms and preventing losses of brain cells. These observations could be further strengthened by conducting behavioral studies and directly assessing seizures via electroencephalography. Moreover, although paraoxon is a potent OP that has been used as a challenge agent in mechanistic studies and in development of OP countermeasures, different OPs apparently differ in their ability to trigger longer-term neurological consequences [84,85]. Future studies involving scL-2PAM as a countermeasure should include testing against other members of the OP family of chemical toxins.

## Data Availability

All data generated or analyzed during this study are available from the corresponding author on reasonable request.
